# Highlighting the role of global longitudinal strain assessment in valvular heart disease

**DOI:** 10.1186/s43044-022-00283-9

**Published:** 2022-05-31

**Authors:** Sidhi Laksono Purwowiyoto, Reynaldo Halomoan

**Affiliations:** 1 Department of Cardiology and Vascular Medicine, RS Pusat Pertamina, Jl. Kyai Maja No.43, RT.4/RW.8, Gunung, Kec. Kby. Baru, Kota Jakarta Selatan, Daerah Khusus Ibukota Jakarta 12120 Indonesia; 2 Faculty of Medicine, Universitas Muhammadiyah Prof. Dr. Hamka, Jl. Raden Patah No.01, RT.002/RW.006, Parung Serab, Kec. Ciledug, Kota Tangerang, Banten 13460 Indonesia; 3 Faculty of Medicine, Universitas Katolik Indonesia Atma Jaya, Jl. Pluit Selatan Raya No.19, RT.21/RW.8, Penjaringan, Kec. Penjaringan, Kota Jkt Utara, Daerah Khusus Ibukota Jakarta 14440 Indonesia

**Keywords:** Global longitudinal strain, Myocardial deformation, Valvular heart disease, Strain imaging

## Abstract

**Background:**

Echocardiography has been the choice for imaging modality for valvular heart disease. It is less invasive, widely available, and allows valvular structure visualization. Echocardiographic assessment often also determines the management. Left ventricular ejection fraction is the most commonly used indicator during echocardiography assessment. It shows signs of left ventricular dysfunction in patients with valve disease. However, most of the time, the ongoing process of cardiac damage may already occur even with preserved cardiac function; further deteriorated ejection fraction will show irreversible cardiac damage. There is a need for a more advanced diagnostic tool to detect early cardiac dysfunction, to prevent further damage.

**Main body:**

Advanced echocardiography imaging using strain imaging allows a physician to evaluate cardiac function more precisely. A more sensitive parameter than left ventricular ejection fraction, global longitudinal strain, can evaluate subclinical myocardial dysfunction before the symptoms occur by evaluating complex cardiac mechanisms. Global longitudinal strain evaluation provides the chance for physicians to determine the intervention needed to prevent further deterioration and permanent cardiac dysfunction. Global longitudinal strain is proven to be beneficial in many types of valvular heart diseases, especially in mitral and aortic valve diseases. It has an excellent diagnostic and prognostic value for patients with valve disease. This review aims to present the superiority of global longitudinal strain compared to left ventricular ejection fraction in assessing cardiac function in patients with valvular heart disease. Clinical usage of global longitudinal strain in several valvular heart diseases is also presented in this review.

**Conclusions:**

The superiority of global longitudinal strain to left ventricular ejection fraction relies on the mechanism where other strains would compensate for the deterioration of longitudinal strain, which is more vulnerable to damage, so the cardiac function is preserved. Therefore, examination of longitudinal strain would give the physician early signs of cardiac function impairment, and prompt management can be conducted.

## Background

Valvular heart diseases (VHD) remain a health burden. In Europe, the prevalence of VHD is around 13.3 million, with aortic stenosis and mitral regurgitation becoming the most common types of VHD [1, 2]. In the US population, about 2.5% population experienced valvular heart disease, which increased in the older age population [3]. It is known that VHD is related to the development of heart failure, especially the moderate and severe one that was found in 14% of patients with heart failure suspicion [4]. The management of VHD is based on the clinical symptoms and evidence of impairment of cardiac function [5, 6]. Imaging examination is essential in evaluating the valve and in determining cardiac dysfunction. Echocardiography is widely available and has an excellent diagnostic value for evaluating cardiac function in patients with the suspected valvular disease [7]. Furthermore, the evaluation of cardiac function by assessing left ventricular ejection fraction (LVEF) can be an essential indicator to determine the need for an invasive management strategy [5, 6, 8]. However, a disruption in myocardial function might already occur even though the ejection fraction is still normal. When the LVEF is already impaired, the myocardial damage may be irreversible [9]. Therefore, an examination to determine early cardiac dysfunction before LVEF impairment may prevent further damage to the myocardial structure. Global longitudinal strain (GLS) is a superior parameter to LVEF because it can be used to note subclinical myocardial dysfunction [10]. GLS also shows good feasibility and is beneficial in evaluating mild cardiac dysfunction [11, 12]. In this review, we aimed to explain the role of GLS assessment in VHD patients and the mechanism.

## Main text

### Strain imaging: general principle and association with myocardial deformation

Strain is an indicator that gives information about any alteration in the length of a segment relative to the baseline length measurement and is presented as a percentage [13]. Myocardial tissue, as a three-dimensional object, has three strains. The analysis of the strain presents myocardial deformation and correlates with stroke volume. Deformation of the left ventricle is affected by three normal strains (longitudinal, circumferential, and radial) and three shear strains (longitudinal–radial, longitudinal–circumferential, and circumferential–radial). The longitudinal strain occurs from the base to the apex when the mitral valve contracts, shown as negative strain. Radial strain is shown as a positive strain value, reflecting the relative thickening of the left ventricular (LV) wall. Lastly, circumferential strain represents the counterclockwise movement of myocardial tissue from base to apex, presented as a negative value. Positive strain defines the thickening, and negative strain value defines shortening. Some factors such as loading, preload, and afterload alteration influence the strain measurement. [13]. Patients with VHD may have changes in LV load that lead to LV geometric alteration. A study by Cramariuc et al. showed that patients with aortic stenosis were associated with a lower myocardial longitudinal deformation even though the LVEF was in the normal range [14]. This finding proved that GLS might provide subclinical cardiac involvement in VHD patients. GLS represents myocardial deformation in the longitudinal plane during the systolic phase [15]. Subendocardial fibers have a role in longitudinal LV contraction, which is affected by increased wall stress [16]. Some studies also pointed out that the myocardial function evaluation by strain evaluation would give an additional value in several valve diseases [17, 18]. Yingchoncharoen et al. also showed that GLS could predict outcomes (death and valve replacement) in asymptomatic aortic stenosis patients with normal ejection fraction (Hazard ratio = 1.14, 95% CI 1.01–1.28, *p* = 0.037) [19].

### Factors affecting strain values in VHD

**Fig. 1:**
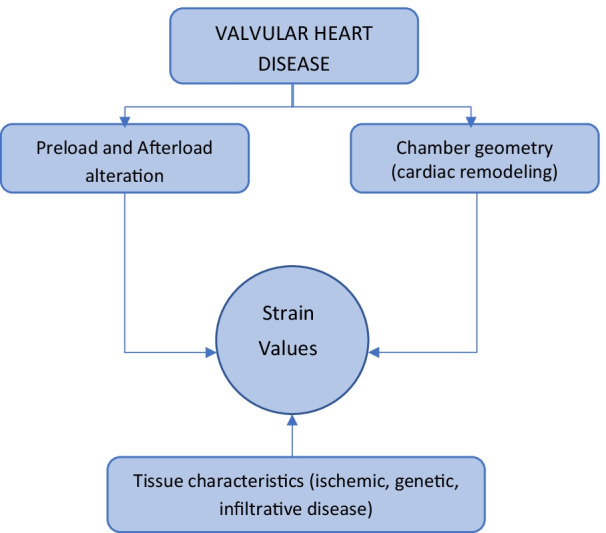
Several factors influence strain values in VHD

Several factors could influence the strain measurement, such as load, structure (geometry), and tissue characteristics (shown in Fig. 1) [20–22].

#### Loading factors

Alteration in both preload and afterload could influence myocardial deformation differently. Increased preload would increase myocardial strain, whereas an increased afterload would decrease myocardial strain [22]. An observational study examined the change in strain related to an acutely decreased preload. The subjects were tilted to reduce the preload, and there was a 25% decrease in the GLS measurement [23]. However, a different phenomenon might occur in chronic preload increase. In the beginning, the strain measurement would increase due to the normal function and became lower because the ventricle started to fail [22, 24]. The systolic strain was also shown to be increased after administration of glyceryl trinitrate sublingually in a study by Burns et al. This study highlighted the role of afterload reduction as the main factor even though both afterload and preload were decreased [20]. The longitudinal strain was also reduced due to an increased afterload in aortic stenosis patients [25].

#### Structure (geometry)

The geometry of the ventricle affects the mechanism of how chamber pressure can be translated into wall stress and how strain can be translated into volume alteration. Patients with VHD may develop cardiac remodeling due to pressure overload. This will lead to the thickening of the walls and decreasing chamber size. These changes are meant to maintain the heart function (assessed by ejection fraction) despite decreasing longitudinal and circumferential strain values [26, 27].

#### Tissue characteristics

Strain values are also determined by the myocardial tissue characteristics such as fibrosis and depositions. Many factors such as ischemic process, cardiotoxicity due to chemotherapy, and the genetic and infiltrative disease may lead to myocardial damage and decrease heart function. The longitudinal strain may be reduced in the early phase because the subendocardial often becomes the first to be affected [22].

### GLS versus LVEF in VHD

LVEF has been one of the several parameters to determine cardiac function and is also recommended to determine the management of valvular heart diseases according to the guideline [5]. It presents both the length and diameter change of the chamber. The length aspect represents longitudinal strain, and the diameter aspect represents circumferential and radial strain [22]. Cardiac load is one of several factors that influences LVEF [28]. In pathological valve conditions, there is a change in cardiac preload and afterload [29]. These conditions become a challenge for assessing LVEF in patients with VHD. LVEF only reflects the relative volume alteration between the end-diastole and end-systole phase and does not assess the myocardial mechanic. Therefore, LVEF has limitations in assessing cardiac function in abnormal hemodynamic conditions [30]. Impaired LVEF often shows a more severe stage of the disease and an irreversible myocardial failure [9, 31].

On the other hand, GLS has the ability to detect any early and subclinical left ventricular dysfunction [32]. GLS measures cardiac function and is not influenced by geometric assumptions [33–35]. Strain can directly evaluate myocardial deformation in a 16-segment model [36]. GLS may become a better parameter than LVEF because it is more sensitive in detecting any alteration in long axis shortening. This sensitivity comes from the vulnerability of the longitudinal strain when damage occurs. Stokke et al. explained that circumferential strain would compensate for the longitudinal strain so that the LVEF can be maintained at a normal value. Therefore, GLS can be used to evaluate early cardiac function in VHD patients when the LVEF is still preserved [26, 37–39]. Furthermore, compared to the two other strains (circumferential and radial), the longitudinal strain is also shown to be more reproducible and applicable in clinical settings [40, 41].

### Clinical application of GLS in valvular heart diseases

GLS becomes a superior parameter compared to LVEF in assessing cardiac function. The European Association of Cardiovascular Imaging (EACVI) and the American Society of Echocardiography (ASE) also acknowledged the benefit of GLS over LVEF [42].

#### Aortic valve diseases

**Fig. 2:**
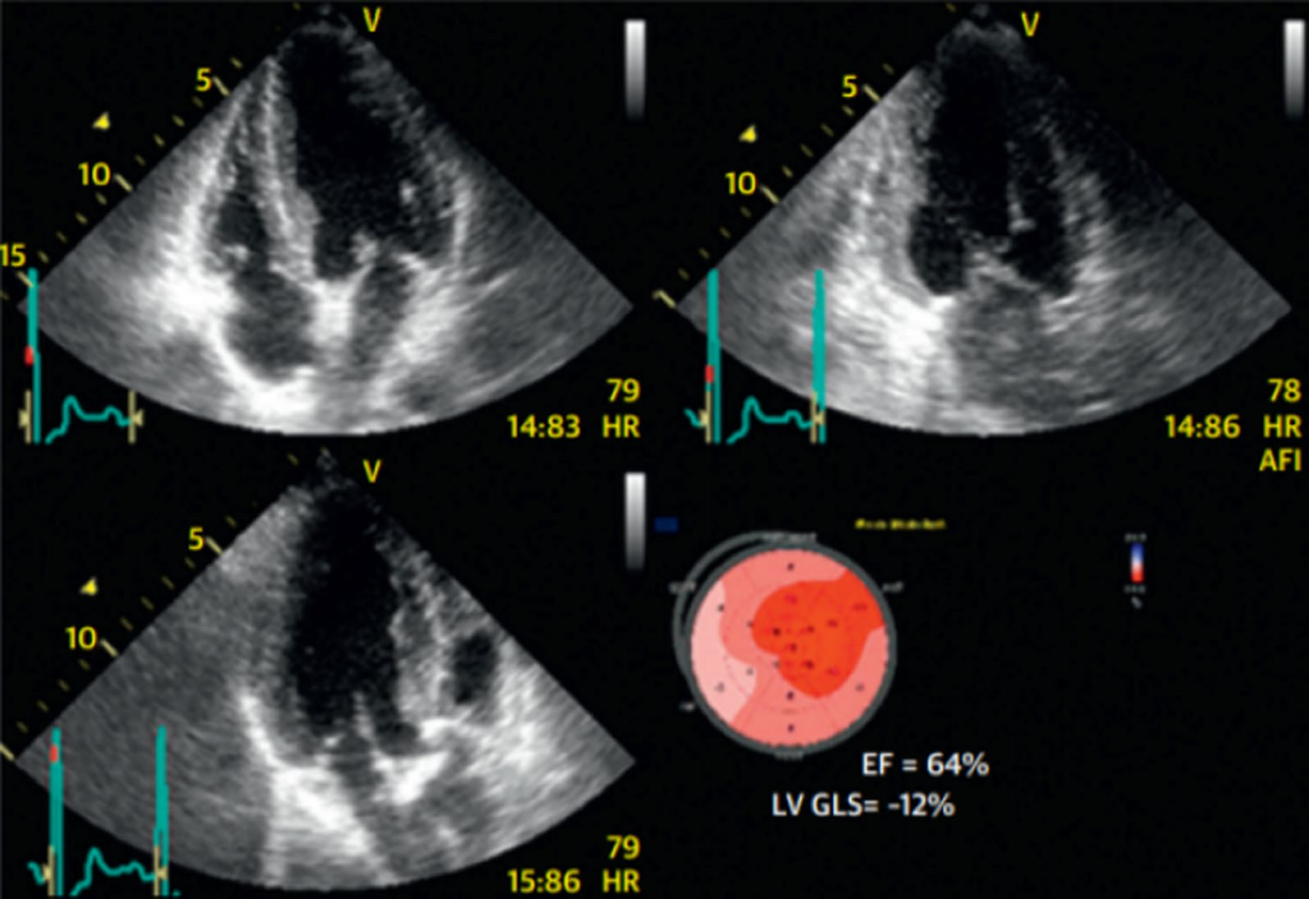
Aortic stenosis patients with normal LVEF but impaired GLS show dysfunction of the left ventricle

Several studies have shown the beneficial use of GLS as a prognostic value in aortic stenosis patients. Vollema et al. evaluated LV GLS in asymptomatic severe aortic stenosis patients with preserved LVEF. This study showed that patients with AS had a significantly impaired LV GLS compared to the control group (mean [SD] LV GLS, − 17.9% [2.5%] vs. − 19.6% [2.1%]; *p* < 0.001) despite the comparable LVEF. The median follow-up (12 months) also showed the more impairment of GLS (mean [SD] LV GLS, − 18.0% [2.6%] to − 16.3% [2.8%]; *p* < 0.001) with unchanged LVEF. These data showed a developing subclinical LV dysfunction over time [43]. These findings were also consistent with the previous study by Lafitte et al. that showed a significant impairment of LV GLS in asymptomatic severe aortic stenosis patients, while there were no changes in the LVEF [44]. Vollema et al. were also able to show that patients who had impaired LV GLS at baseline had a higher risk of developing symptoms and required interventional therapy compared to patients with preserved LV GLS [43]. These findings could determine the need for valve intervention before the deterioration of LVEF to prevent irreversible cardiac remodeling (e.g., myocardial fibrosis) [43, 45]. LV GLS measurement is also useful as a predictor of mortality. Kusunoese et al. showed that LV GLS was an independent predictor of mortality (hazard ratio [HR], 1.05; 1.03–1.07; *p* < 0.001) [46]. Ng et al. found an independent relation between LV GLS and all-cause mortality. Individuals with severe AS who had normal LVEF but abnormal LV GLS had the similar poor long prognosis as patients with severe AS who had impaired LVEF. Patients with abnormal LV GLS had a higher mortality risk, regardless of LVEF or AS severity. This may have relevance for the appropriate time to replace an aortic valve in individuals with severe AS. This study also found that patients with severe aortic stenosis with subclinical cardiac dysfunction had higher mortality than the patients with stable LV GLS [47]. Example of decreased GLS in AS patients with normal LVEF is presented below (shown in Fig. 2).

GLS measurement in aortic regurgitation is less studied. However, several studies were able to show the role of GLS in aortic regurgitation patients. A study by Alashi et al. showed that lower LV GLS had a prognostic value for mortality in the long term. The study was conducted on asymptomatic patients with aortic regurgitation and preserved cardiac function. The mortality risk at 5 years was also increased if the LV GLS value was lower than − 19.5%. In addition, according to the findings of this study, LV GLS has both incremental and additive prognostic value. Using LV GLS might provide a synergistic improvement in risk reclassification in individuals with severe MR prior to the development of overt LV systolic failure or symptoms. As a result, LV GLS might assist as basis for further optimizing treatments in asymptomatic individuals before to the development of atrial fibrillation or pulmonary hypertension [48]. Another study presented the data on symptomatic versus asymptomatic aortic regurgitation patients. The value of LV GLS in symptomatic patients was lower than in the asymptomatic patients (− 14.9 ± 3.0% vs. − 16.8 ± 2.5%, *p* < 0.001) [49]. This study also found that of all asymptomatic patients, some of the patients who were indicated for surgery had more impaired LV GLS values [39].

#### Mitral valve diseases

Mitral regurgitation is a common valvular disease globally and can be classified into primary and secondary mitral regurgitation [49]. In primary mitral regurgitation, cardiac dysfunction may not be shown by an impaired LVEF. The absence of afterload causes a condition of hyperdynamic LVEF, so the LVEF may still be normal even though the myocardial starts to deteriorate [10]. Many studies examined the use of LV GLS measurement as an outcome predictor after surgery and associated with mortality. Mentias et al., in their study, examined 737 patients with asymptomatic primary severe mitral regurgitation with preserved cardiac function. In this study, LV GLS <  − 21.7% was associated with mortality [24]. However, the cutoff of the GLS value in the study was slightly higher than the lower limit of normal. This might suggest that in primary mitral regurgitation, the GLS value, which was considered normal, had already been related to a worse outcome [30]. Mascle et al. studied the role of preoperative GLS as a predictor value of postoperative LVEF. This study showed that patients with postoperative LVEF < 50% had worse preoperative GLS than the patients with LVEF at least 50% postoperatively (− 17.0% ± 2.8% vs. − 19.6% ± 3.6%, *p* < 0.01). However, the preoperative LVEF measurement showed no differences [50]. A study by Alashi et al. also showed that preoperative GLS was an independent predictor for postoperative impaired LVEF (< 50%) and all-cause mortality [51].

In secondary mitral regurgitation, GLS is also superior to LVEF in showing cardiac function. Kamperidis et al. showed that patients with severe secondary mitral regurgitation had more impaired GLS than the 'none or less than mild' secondary mitral regurgitation patients with comparable LVEF [52]. The study on the evaluation of mitral stenosis by GLS is limited. However, a study by Gerede et al. was able to show the association of LV GLS with the progression of mitral stenosis. In this study, patients with a GLS value worse than − 16.98% had a more progressive condition [53]. This study showed that GLS measurement could be done to evaluate the progression of mitral stenosis.

#### Other valve diseases

There are very limited studies about GLS application in tricuspid and pulmonary valve disease. However, several studies were able to show the benefit of GLS measurement. Right ventricular (RV) longitudinal strain was superior to other echocardiographic measurements and was related to outcome in patients with tricuspid regurgitation [54]. In pulmonary valve disease, preintervention RV longitudinal strain was used as a predictor of function after valve intervention [55]. Studies of GLS application in VHD are shown in Table 1.

**Table 1:** Summary of studies showing GLS application in valvular heart disease

References	*n*	Patients	Method	Clinical application and GLS cutoff
*Aortic stenosis*				
Vollema et al. [43]	220	Asymptomatic patients with severe AS	Interobserver	LV GLS was impaired despite comparable LVEF, cutoff − 18.2%
Lafitte et al. [44]	65	Asymptomatic patients with severe AS	Intraobserver	To evaluate exercise tolerance and patients' outcomes, cutoff: − 18%
Kusunose et al. [46]	395	Patients with severe AS preserved LVEF	Intraobserver and interobserver	LV GLS gave an additional prognostic value and predicts mortality in moderate–severe and severe AS patients, cutoff: N/A
Ng et al. [47]	688	Patients with mild, moderate, and severe AS	Intraobserver and interobserver	LV GLS may be used to stratify the risk in severe AS patients and may affect the timing of valve replacement, cutoff − 14%
*Aortic regurgitation*				
Alashi et al. [48]	1063	Patients with chronic aortic regurgitation and preserved LVEF	Intraobserver and interobserver	LV GLS was related to longer-term mortality despite preserved ejection fraction, cutoff − 19.5%
Ewe et al. [39]	129	Patients with moderate–severe or severe AR with preserved LVEF	interobserver	In patients with asymptomatic AR, GLS may identify the needs for surgery during follow-up, cutoff − 17.4%
*Mitral regurgitation*				
Mentias et al. ^24^	737	Patients with asymptomatic primary MR and preserved LVEF	Intraobserver and interobserver	Resting LV GLS was related to mortality, cutoff − 21%
Alashi et al. [51]	448	Patients with asymptomatic MR and preserved LVEF	Intraobserver and interobserver	LV GLS gave an incremental value for risk stratification, cutoff: N/A
Kamperidis et al. [52]	150	Patients with severe secondary MR and none or less than mild MR	Intraobserver and interobserver	LV GLS was better than LVEF in showing LV dysfunction, cutoff: N/A
*Mitral stenosis*				
Gerede et al. [53]	48	Patients with mild-to-moderate MS	Intraobserver	GLS was able to predict MS progression, cutoff − 16.98%

AR, aortic regurgitation; AS, aortic stenosis; LVEF, left ventricular ejection fraction; LV GLS, left ventricle global longitudinal strain; MR, mitral regurgitation; MS, mitral stenosis

**Fig. 3:**
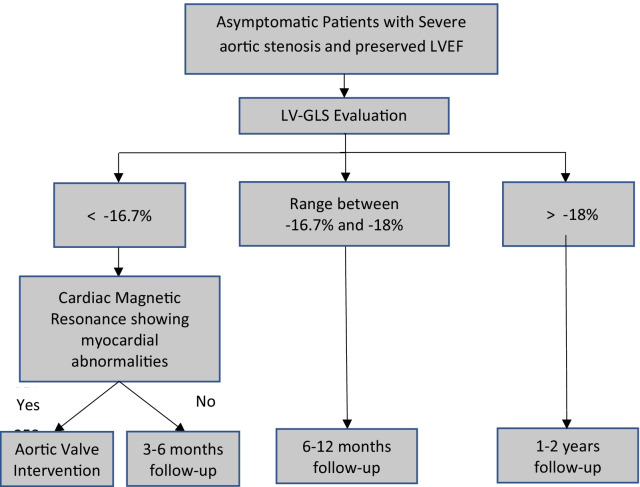
Algorithm for asymptomatic patients with severe aortic stenosis

There is no consensus on the standard GLS value used as a benchmark for determining left ventricular dysfunction in patients with VHD. However, Dahl et al. proposed a new algorithm for asymptomatic patients with severe aortic stenosis (Fig. 3). Further algorithms for other valve diseases are needed [56].

### Conclusion

Assessment of global longitudinal strain in valvular heart disease is beneficial and superior to LVEF because of the ability to detect cardiac dysfunction in asymptomatic patients due to compensation by other groups of strain. This advantage of GLS can be further used to determine the therapeutic strategy for the patients. GLS has been studied in many mitral and aortic valve diseases. Further studies are needed to establish the role of GLS in various tricuspid and pulmonary valve diseases.

## Data Availability

Not applicable.

## Abbreviations

VHD: Valvular heart disease
LVEF: Left ventricular ejection fraction
GLS: Global longitudinal strain
LV: Left ventricle
EACVI: European Association of Cardiovascular Imaging
ASE: American Society of Echocardiography
RV: Right ventricular
